# Protease and chitinase activity of *Trichoderma* isolates and their synergy with biochar in enhancing chickpea defense related enzymes

**DOI:** 10.3389/fmicb.2025.1699251

**Published:** 2025-12-10

**Authors:** Ranjna Kumari, Bhupendra Koul, Vipul Kumar, Adesh Kumar, Manpreet Kaur Somal, Rohan Samir Kumar Sachan

**Affiliations:** 1 Department of Botany, Lovely Professional University, Phagwara, Punjab, India; 2 Department of Biotechnology, Lovely Professional University, Phagwara, Punjab, India; 3 Department of Plant Pathology, School of Agriculture, Lovely Professional University, Phagwara, Punjab, India; 4 Department of Biotechnology, Chandigarh School of Business, CGC Jhanjeri, Mohali, Punjab, India

**Keywords:** biocontrol, elicitor, hydrolytic enzymes, pathogen suppression, plant immunity

## Abstract

**Background:**

Soil-borne pathogens such as *Sclerotium rolfsii* (*Agroathelia rolfsii* Sacc.) and *Fusarium oxysporum* f. sp. *ciceri* pose serious threats to chickpea (*Cicer arietinum* L.) production. *Trichoderma* spp. are widely recognized in modern agriculture as effective biocontrol agents due to their ability to produce several lytic enzymes, including chitinases, glucanases and proteases, which contribute to the inhibition of plant pathogens.

**Objectives:**

This study aimed to screen *Trichoderma* isolates for protease and chitinase activity, evaluate their antagonistic potential against two chickpea pathogens, and assess the synergistic effects of *Trichoderma* and biochar in disease suppression and plant growth promotion. This study investigated the protease and chitinase activities of different *Trichoderma* isolates and evaluated their synergistic potential with biochar in promoting defense-related enzymes in chickpea (*Cicer arietinum* L.).

**Methods:**

A total of 21 *Trichoderma* isolates were screened for protease and chitinase activity. Four potent strains—*T. harzianum* (PBT13), *T. virens* (PBT3), *T. lixii* (PBT14), and *T. asperellum* (PBT4)—were selected for further evaluation. Antagonistic activity against *F. oxysporum* f. sp. *ciceri* and *S. rolfsii* was assessed using dual culture assays and scanning electron microscopy (SEM). The extracellular chitinase activity of the most active strain was quantified, and its inhibitory effect on pathogenic growth was determined. The combined application of *T. harzianum* and rice husk biochar significantly influenced disease incidence, defense enzyme activity, germination, chlorophyll content, sclerotia formation, and *Trichoderma* survivability under greenhouse and field conditions.

**Results:**

Among the tested isolates, *T. harzianum* (PBT13) showed the highest enzymatic activity and strong antagonism against both pathogens. Extracellular chitinase activity peaked at 60 U/mL, suppressing *in vitro* growth of *F. oxysporum* f. sp. *ciceri* by 95.95% and *S. rolfsii* by 97.10%. Greenhouse/field trials revealed that combining *T. harzianum* with rice husk biochar significantly reduced disease incidence, enhanced plant defense, enzyme activity, improved germination and chlorophyll content, reduced sclerotia formation, and promoted *Trichoderma* survival in soil.

**Conclusion:**

The study demonstrates that enzyme-active *Trichoderma* strains, particularly *T. harzianum* (PBT13), in combination with rice husk biochar, provides a sustainable and synergistic approach for managing soil-borne diseases in chickpea. This integrated strategy not only suppresses pathogens but also improves plant health and resilience, offering a viable alternative to chemical fungicides.

## 1 Introduction

Chickpea (*Cicer arietinum* L.) is a globally significant pulse crop, valued for its high nutritional content, including proteins (18–20%), fat (5%), carbohydrates (60%), and dietary fibres (10–20%) (Jukanti et al., 2012; Kumar et al., 2025). However, climate change and other stresses, (biotic and abiotic), constantly hamper the crop production (Landi et al., 2019). Approximately 50% of crop losses are attributed to abiotic stress and the figure is steadily rising due to ongoing climate change (Minhas et al., 2017). However, biotic stresses in plants arise mainly from pathogens and pests (Gimenez et al., 2018). Globally, pests reduce annual agricultural productivity by an estimated 18 to 25% (Poveda, 2021), while pathogens inflict 10 to 15% of total crop losses (Mohammad-Razdari et al., 2022). Among pests and pathogens, the major threats to chickpea crops and their associated losses are: nematodes: 13–40% (Zwart et al., 2019), viruses: 10–60% (Kumar and Gupta, 2019), pod borers: 10–40% (Said et al., 2022), aphids and other insects: 10–30% (Jaba et al., 2021), and fungal phytopathogens: 50–60% (Pande et al., 2023), all causing substantial yield losses (Mejía et al., 2021). Among these drivers of crop loss, fungal pathogens, particularly soil-borne fungi, such as *Fusarium oxysporum*, *Rhizoctonia bataticola*, and *Sclerotium rolfsii*, have been reported as a major threat, causing significant crop losses (Sharma et al., 2017). Collar rot caused by *S. rolfsii* is devastating, resulting in 10–30% yield losses annually and up to 95% seedling mortality (Chowdary et al., 2024; Haware et al., 1996). This necrotrophic fungus survives in soil through resilient sclerotia and produces various cell wall-degrading enzymes (CWDEs), such as oxalic acid, cellulases, and pectinases that facilitate rapid colonization of host tissue (Kubicek et al., 2014; Punja and Jenkins, 1984). The persistent nature of these soil-borne pathogens due to long-lived structures like sclerotia and chlamydospores poses a major challenge to effective disease management (Koma, 2023; Elad and Chet, 1995). To overcome these challenges, both chemical fungicides and biocontrol agents are commonly used. However, chemical fungicides often provide limited and inconsistent results (Mandal et al., 2009), and may contribute to environmental pollution and resistance development, whereas biocontrol agents are eco-friendly, non-hazardous, and offer more sustainable solutions (Manhas and Kaur, 2016; Sharma et al., 2020). Hence, there is an increasing shift toward Integrated Disease Management (IDM) strategies combining biological control, organic amendments, and cultural practices to achieve sustainable disease protection (Bhuiyan, 2017). *Trichoderma* spp. secretes extracellular lytic enzymes, including chitinases, *β*-glucanases, and proteases, which are generated during interactions with the cell walls of pathogens, effectively degrading them (Wonglom et al., 2019; Baiyee et al., 2019). Although other enzymes may participate in the comprehensive breakdown of fungal infections’ cell walls, chitinase is predominantly regarded as the principal enzyme due to its substrate, chitin, being the most prevalent constituent in the cell walls of numerous fungal species (Hartl et al., 2012; Baek et al., 1999). At present, chitin degradation and chitinases are playing an important role in a wide variety of biological and biotechnological processes, ranging from the exploitation and environmental clean-up of chitinous wastes to plant defense systems and biological control (Loc et al., 2020). Chitinases are found in various organisms, including fungi, bacteria, yeasts, plants, and actinomycetes (Hamid et al., 2013). Research on the characterization and activity of extracellular chitinase from *Trichoderma* species has been conducted for an extended period, including studies on *T. harzianum* (Ulhoa and Peberdy, 1991; El-Katatny et al., 2000, 2001; Sandhya et al., 2004), *T. harzianum* (Lima et al., 1999), and *T. virens* (Baek et al., 1999). Species like *T. harzianum*, *T. virens*, *T. asperellum*, and *T. lixii* exhibit strong antagonistic activity through mechanisms such as mycoparasitism, nutrient competition, production of CWDEs (chitinases, *β*-1,3-glucanases, and proteases), and synthesis of secondary metabolites, including gliotoxin, 6-pentyl-2H-pyran-2-one, and harzianolide. Thus, they are widely used due to their broad-spectrum antagonistic properties (Sharma et al., 2002; Singh et al., 2003). Chitinase and protease are particularly critical as they degrade the structural components of fungal cell walls, primarily glycoproteins and chitin (Lorito et al., 1994). SEM studies have demonstrated *Trichoderma*’s hyphal coiling, penetration, and lysis of *S. rolfsii* and *F. oxysporum* hyphae (Supplementary Figure 1), confirming its mycoparasitic activity (Tyśkiewicz et al., 2022; Dutta et al., 2023; Kumari et al., 2025). The integration of *Trichoderma* with organic soil amendments, such as biochar (BC), has shown promise as a promising avenue for enhancing plant health, soil resilience, and disease suppression. Biochar, a carbon-rich by-product of pyrolysis, improves soil structure, cation exchange capacity, pH, and microbial habitat (Lehmann et al., 2011; Pandao et al., 2023). Its porous structure enhances colonization and activity of beneficial microbes and can indirectly suppress pathogens by modifying the soil environment and reducing sclerotia formation (Ameloot et al., 2014; Hale et al., 2015). Moreover, biochar influences the soil microbial community, enzyme activities, and nutrient cycling factors critical for sustainable plant growth (Trivedi et al., 2020). Studies have shown that combining *Trichoderma* spp. with biochar can enhance plant defense responses by upregulating enzymes such as peroxidase, catalase, and phenylalanine ammonia-lyase (PAL), leading to improved systemic resistance in plants (Farhangi-Abriz and Torabian, 2017; Shoaib et al., 2013; Mitter et al., 2019). These interactions have been associated with improved seed germination, chlorophyll content, root establishment, and suppression of soil-borne pathogens (Marinari et al., 2000; Liu et al., 2020). Given the multifunctional role of hydrolytic enzymes in fungal suppression and the soil-enhancing properties of biochar, the evaluation of protease and chitinase activity among *Trichoderma* strains, alongside their interactions with biochar, represents an ecologically sound and sustainable strategy for managing collar rot and other soil-borne diseases in chickpea. The involvement of lytic enzymes and antibiotics from *Trichoderma* in the control of plant diseases has been examined in several experimental investigations and reviews. However, a full assessment of extracellular enzymes from *Trichoderma* and its function in plant disease management and the induction of immunity has not been extensively investigated. This study focuses on the role of *Trichoderma*-derived extracellular enzymes in suppressing plant pathogens and enhancing plant immune responses, along with the synergistic effect of *Trichoderma* and biochar for Sclerotium rot and *Fusarium* wilt.

## 2 Materials and methods

### 2.1 Sampling, isolation, and identification of *Trichoderma* strains

**Figure 1:**
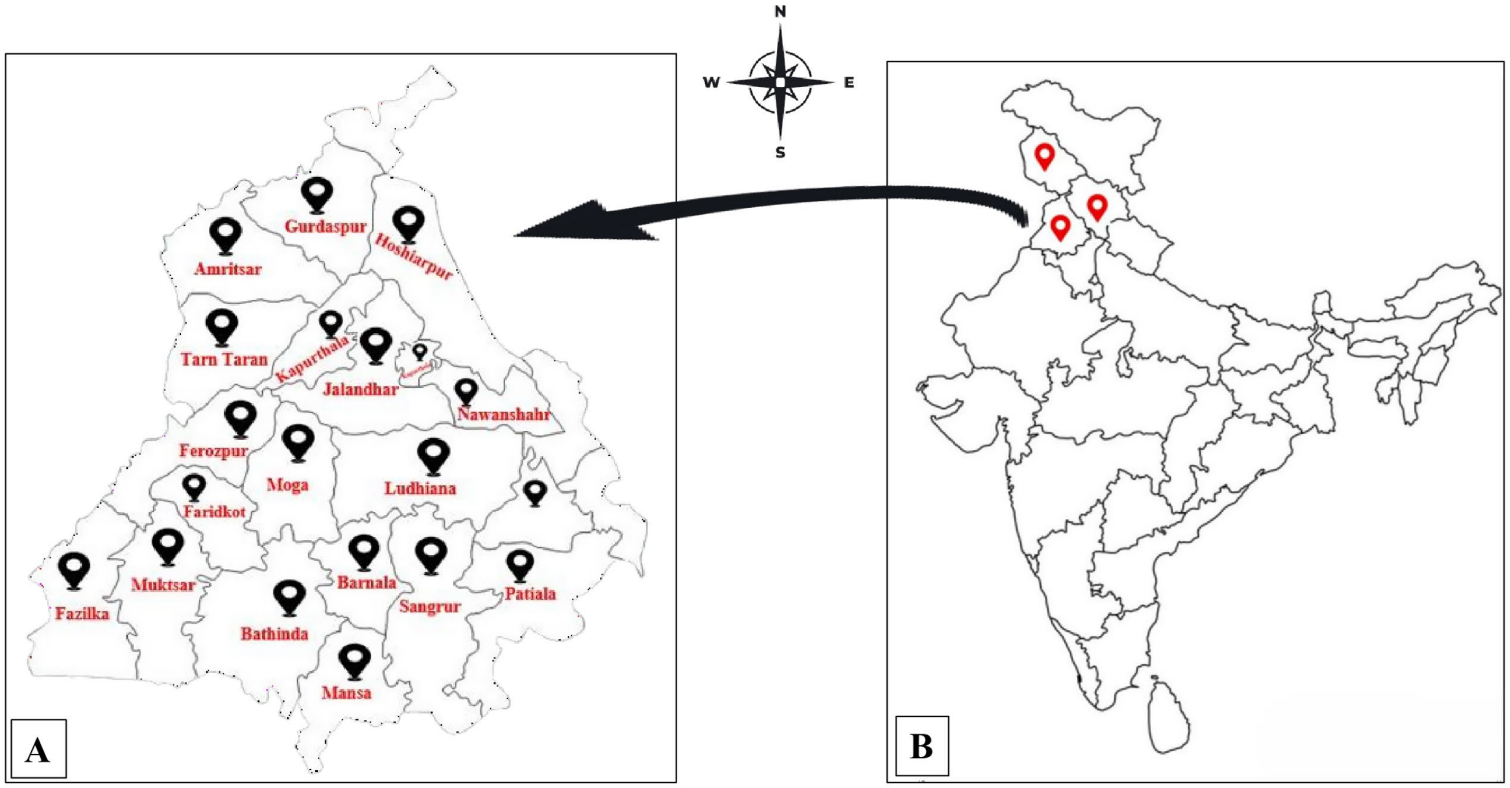
Soil sample collection. **(A)** Sample collection sites in Punjab. **(B)** Map of India showing the three main locations of sample collection.

Twenty-one *Trichoderma* isolates (Jammu and Kashmir: 32.99059° N, 74.93717° E; Mansa: 29.9995° N, 75.3937° E; Bathinda: 30.2110° N, 74.9455° E; Muktsar: 30.4762° N, 74.5122° E; Fazilka: 30.4036° N, 74.0280° E; Faridkot: 30.6774° N, 74.7539° E; Ferozpur: 30.9331° N, 74.6225° E; Moga: 30.8230° N, 75.1734° E; Barnala: 30.8230° N, 75.1734° E; Sangrur: 30.8230° N, 75.1734° E; Patiala: 30.3398° N, 76.3869° E; Malerkotla: 30.5246° N, 75.8783° E; Ludhiana: 30.9010° N, 75.8573° E; Jalandhar: 31.3260° N, 75.5762° E; Fatehpur Sahib 30.6435° N, 76.3970° E; Tarn Taran 31.4539° N, 74.9268° E; Amritsar: 31.6340° N, 74.8723° E; Gurdaspur: 32.0414° N, 75.4031° E; Kapurthala: 31.3723° N, 75.4018° E; Nawanshahr: 31.1256° N, 76.1186° E; Himachal Pradesh: 32.1024° N, 77.5619° E) were obtained from rhizospheric soils collected across Punjab, Jammu and Kashmir and Himachal Pradesh, India (Figure 1). Samples were stored in sterilized zip-lock bags at 4 °C. One gram of soil was serially diluted to 10^−4^, and 100 μL aliquots were spread on Rose Bengal agar (RBA) for isolation. Morphologically distinct colonies were purified on PDA using the single-spore technique (O’Gorman et al., 2009) and kept on PDA slants at 4 °C. Isolates were screened for biocontrol efficacy (mycelial inhibition, hydrolytic enzyme production, growth rate) against *Sclerotium rolfsii* and *F. oxysporum*. Four potent isolates were identified through ITS analysis and partial genome sequencing (Kumari et al., 2024), and these findings were later published (Kumari et al., 2025).

### 2.2 Multiplication of pathogen inoculum

The pathogens (*Sclerotium rolfsii* and *Fusarium oxysporum*) utilized in this study were sourced from the Indian Type Culture Collection (ITCC), IARI, New Delhi, India, under the accession numbers: ITCC No. 8527 and ITCC No. 6341, respectively. Pathogens were mass multiplied on sorghum grains following the method prescribed by Sennoi et al. (2012). Sorghum grains (250 g) were soaked in tap water overnight. Approximately 100 g of soaked grains were filled in an autoclavable polybag and tied with a thread, and subsequently sterilized for 20 min at 121 °C in an autoclave. After sterilization, the grains were inoculated with two mycelial bits of the pathogen obtained from a five-day-old culture of *Sclerotium rolfsii* and *Fusarium oxysporum*. The inoculated bags were kept in a BOD incubator set at 28 ± 2 °C for 1 week (Sennoi et al., 2012).

### 2.3 Pathogenicity test

Pathogenicity test was carried out under controlled pot culture condition during 2023–2024 cropping season. The field soil was pasteurized in an autoclave at 121.6 °C for 20 min on three successive days to eliminate all the contaminants. The sick pots were prepared by adding 25 g of prepared inoculum into each earthen pot. Five healthy chickpea seeds (variety PBG 7) were treated with 1% sodium hypochlorite solution and sown in each pot. These experiments were repeated three times. The pots without inoculum were considered as a control. Typical symptoms of the disease were observed after 90 days, and PDI% (percent disease incidence) was measured using the equation (Kokalis-Burelle et al., 1997). The pathogen was re-isolated from the affected plants and subsequently compared with the original culture after harvest.


$$\mathrm{PDI}(\%)=\frac{\text{Number of diseased plants}}{\text{Total plants observed}}\times 100$$

### 2.4 Screening of protease and chitinase activity

#### 2.4.1 Qualitative assay

The enzyme activity of *Trichoderma* isolates was tested using an agar plate assay with specific media: CAM (Casein Agar Medium) and CDM (Chitinase Detection Medium) for protease and chitinase activity, respectively. Both media were sterilized and poured into separate Petri plates. One *Trichoderma* disc (6 mm) was placed in the centre of each Petri dish. The inoculated plates were kept in a BOD incubator set at 26 ± 2 °C for 3 to 5 days. After 5 days, the Petri dishes were treated with green dye (Bromocresol) to detect proteolytic activity, specified by transparent zone formation around the colonies, surrounded by a greenish-blue area due to the medium’s pH (8.0 ± 0.2). The colourless zones indicated protease activity, contrasting with the rest of the plate’s greenish-blue colour. For chitinase activity, chitinase production was indicated by the formation of a purple-colored zone around colonies, enhanced by bromo cresol purple binding to unhydrolyzed protein. Zone diameter and color intensity were used to evaluate chitinase activity. This experiment was repeated three times (Cherkupally et al., 2017).

#### 2.4.2 Quantitative assay

##### 2.4.2.1 Preparation of enzyme source

A 3 mm disc of *Trichoderma* strain(s) was inoculated into an Erlenmeyer flask containing TLE medium. The medium contained, 0.3 g of calcium chloride dihydrate (CaCl₂·2H₂O), 2.0 g of monopotassium phosphate (KH₂PO₄), 1.4 g of ammonium sulphate (NH₄)₂SO₄, 0.3 g of magnesium sulphate heptahydrate (MgSO₄·7H₂O), 0.3 g of urea 0.3 g, 1.0 g of peptone, and 0.1% micronutrients (Zn^2+^, Fe^2+^, Cu^2+^, Mn^2+^) 0.1%. The cell wall of *Sclerotium rolfsii* (0.5%) was utilized as a source of carbon and nitrogen for enzyme production by *Trichoderma* after lyophilization. The inoculated conical flasks were kept in a BOD incubator-cum shaker set at 28 ± 2 °C, 120 rpm for 48 h to ensure continuous agitation. After 48 h, the fungal biomass was separated by filter paper, and the resulting extracts were utilized as the enzyme source (Geraldine et al., 2013).

##### 2.4.2.2 Estimation of the protease enzyme

Protease activity was estimated using 0.25% (w/v) azocasein as the substrate, dissolved in phosphate–citrate buffer (50 mM) adjusted to pH 5.0. The assay mixture consisted of buffer solution (40 μL), enzyme extract (20 μL), and azocasein (40 μL) as a substrate. The reaction mixture was incubated at 37 °C for 30 min, after which 100 μL of 10% (w/v) trichloroacetic acid (TCA) was added to terminate the reaction. The samples were subsequently incubated at 4 °C for 10 min. After centrifugation (698.75 g for 30 min), supernatant (100 μL) was decanted into a microplate and mixed with 1 M sodium hydroxide (100 μL), and optical density (OD) was recorded at 450 nm. One unit of protease enzyme activity is defined as the quantity of enzyme that induces an increase in optical density per minute. The protein concentration was quantified utilizing the Bradford method (Bradford, 1976), employing BSA (bovine serum albumin) as the standard (Geraldine et al., 2013).

##### 2.4.2.3 Estimation of chitinase enzyme

Chitinase extraction was conducted using the following procedure, explained by Tiwari et al. (2024). For the enzyme assay, 1.5% colloidal chitin was incorporated into the Czapek Dox Broth medium as a supplement. After the preparation of the medium, two bits (7 mm) of *Trichoderma* were inoculated and placed in a BOD incubator at 28 ± 2 °C for 6–8 days. The liquid culture was collected at 4-day and 8-day intervals and centrifuged at 5,000 rpm for 10 min. The resulting supernatant was harvested and utilized for the chitinase assay. The protein content in the samples was quantified by a spectrophotometer at 595 nm, using bovine serum albumin (BSA) as the reference standard. Chitinase activity was presented in μg/mL, representing the production of the enzyme by the *Trichoderma* strains (Tiwari et al., 2024).

### 2.5 *In vitro* antipathogenic property of chitinase

An assay was performed to evaluate the efficacy of chitinase in inhibiting hyphal growth to determine its effectiveness against the fungus. Proliferation of the phytopathogenic fungi *S. rolfsii* and *Fusarium* sp., both characterized by the presence of chitin in their cell walls. A 90 mm × 15 mm petri plate containing 1/2 potato dextrose (PD) broth (potato dextrose agar (PDA) without agar) was augmented with 10–60 U/mL of enzyme and 10 μL of fungal spore suspension (about 106 spores/mL). The culture was maintained at 28 °C for 36 to 48 h to monitor fungal proliferation. A centrifuge was utilized to isolate the mycelium cells at 4,000 rpm for 5 min, and subsequently rinsed with distilled water to acquire the fresh biomass. The fresh biomass was desiccated at 65 °C until a stable weight was attained to ascertain the dry biomass (Loc et al., 2020).

### 2.6 Plant material

The chickpea variety utilized was PBG 7, a desi-type known for its modest yield of 8 quintal/acre was highly susceptible to *Fusarium oxysporum* f. sp. *ciceri* and *Sclerotium rolfsii* in Indian agro-climatic settings. We obtained certified seeds of *C. arietinum* (PBG 7) from Punjab Agricultural University (PAU), Ludhiana (30.9017° N and longitude 75.8053° E), India. This was executed to ensure the plants exhibited genetic consistency and optimal physiological quality. Before sowing and treatment, the seedlings were surface sterilized to eliminate epiphytic microbial pollutants that could interfere with microbial inoculation or subsequent enzymatic assays. The seeds were submerged in 1% (v/v) sodium hypochlorite solution in a conical flask with continuous agitation for 3 min (Taylor et al., 1998). Subsequently, the seeds were rinsed thrice with sterile distilled water (DW) to eliminate any residual sterilant. The seeds were subsequently air-dried in a laminar airflow (LAF) cabinet for approximately 30 min under sterile conditions. This was executed to provide uniform drying and facilitate handling for biopriming procedures. The seed sterilization procedure was carried out according to protocols validated for legumes (Singh et al., 2019). This ensured optimal conditions for microbial colonization during *Trichoderma* biopriming while preserving seed viability. Meticulous preparation of the host material was essential to ensure that the results of the interaction tests between biochar *Trichoderma harzianum*, and the host plant under biotic stress from soil-borne pathogens were consistent.

### 2.7 Treatment combinations

The experiment was conducted using a completely randomized design (CRD) with three replications to assess the various treatment combinations. The treatments included: T1—*Fusarium oxysporum* f. sp. *ciceri* (FOC), T2—*Sclerotium rolfsii* (SR), T3—*Trichoderma harzianum* alone, T4—FOC with biochar, T5—SR with biochar, T6—FOC with *T. harzianum*, T7—SR with *T. harzianum*, T8—FOC with biochar and *T. harzianum*, and T9—SR with biochar and *T. harzianum*. All pathogen-associated treatments were performed with either FOC or SR as previously described. This experimental setup facilitated the assessment of both preventive and suppressive effects of *T. harzianum* and biochar, individually as well as in combination.

### 2.8 Assays of defense-related enzymes

#### 2.8.1 Phenylalanine ammonia-lyase assay

The activity of phenylalanine ammonia-lyase (PAL; EC 4.3.1.5) was estimated to determine whether the phenylpropanoid pathway, crucial for plant defense, was activated or not. 5 mL of 0.1 M sodium borate buffer (pH 8.8), including 1 mM EDTA and 1% PVP, was employed to homogenize 0.5 g of fresh chickpea root tissue. This inhibited phenolic oxidation. The homogenate was centrifuged (12,000 rpm for 15 min at 4 °C), and the resulting supernatant was utilized as the enzyme extract. The assessment of PAL activity was conducted following the methodology of Dickerson et al. (1984), which involved assessing the synthesis of trans-cinnamic acid from L-phenylalanine. The reaction mixture consisted of 1.5 mL of 0.1 M Tris–HCl buffer (pH 8.8), 0.5 mL of 10 mM L-phenylalanine, and 0.5 mL of enzyme extract. The reaction mixture was incubated at 37 °C for 1 h, after which trans-cinnamic acid production was spectrophotometrically measured at 290 nm. Phenylalanine ammonia-lyase (PAL) activity was expressed as μmol of trans-cinnamic acid formed per minute per gram of fresh weight (FW) (Dickerson et al., 1984; Jameel et al., 2021).

#### 2.8.2 Catalase assay

The catalase (CAT) activity in chickpea root tissues to evaluate the efficacy of hydrogen peroxide (H₂O₂) detoxification in the presence of living organisms. 0.5 g of fresh root sample was crushed in 5 mL of ice-cold 0.1 M sodium phosphate buffer (pH 7.0), contain 1 mM EDTA and 1% polyvinylpyrrolidone (PVP) to maintain enzyme stability. The homogenate was centrifuged at 12,000 rpm for 15 min at 4 °C, and the supernatant was utilized to extract the enzymes. The methodology of Aebi (1984) was followed to assess the CAT activity. It involved measuring the decomposition rate of H₂O₂ spectrophotometrically at 240 nm. The reaction mixture contained 2.5 mL of 50 mM phosphate buffer (pH 7.0), 0.4 mL of 15 mM H₂O₂, and 0.1 mL of enzyme extract. The reduction in absorbance per minute was quantified and the enzyme activity was expressed as μmol H₂O₂ decomposed per minute per gram of fresh weight (FW) (Aebi, 1984).

#### 2.8.3 Peroxidase assay

Peroxidase (POD) assay was performed to evaluate the oxidative defense response in treated chickpea plants. Root tissues (0.5 g) were homogenized in 5 mL of ice-cold 0.1 M sodium phosphate buffer (pH 7.0) containing 1 mM EDTA and 1% PVP. The homogenate was centrifuged at 12,000 rpm for 15 min at 4 °C, and the supernatant was used as the enzyme source. POD activity was assayed following the protocol of Chance and Maehly (1955), using guaiacol as the electron donor. The reaction mixture comprised 1.5 mL of 0.05 M phosphate buffer (pH 6.5), 1.0 mL of 20 mM guaiacol, 0.3 mL of 40 mM H₂O₂, and 0.2 mL of enzyme extract. The increase in absorbance at 470 nm, due to the formation of tetraguaiacol, was recorded for one minute. POD activity was calculated as the change in absorbance per minute per gram of fresh weight (FW) (Chance and Maehly, 1955).

### 2.9 Disease assessment

Disease incidence was calculated 40 days after inoculation as a percentage of diseased plants in the treatment by using the following formula


$$\begin{array}{l} \text{Disease incidence}\;(\%)=\text{Number of diseased plants}\times 100/\\ \text{Total number of plants} \end{array}$$

The following formula was used to compute the percentage provided by biochar, *Trichoderma* and combined application, where *A* represents the PDI of untreated control plants and *B* represents the PDI of treated plants (Attia et al., 2022).


Protection(%)=A−B×100/A

## 3 Results

### 3.1 Sampling, isolation, and identification of *Trichoderma* strains

Distinct fungal colonies were recovered from soil samples, and *Trichoderma* isolates were successfully identified through morphological and molecular characterization (Kumari et al., 2024, 2025).

### 3.2 Pathogenicity test

Pathogenicity test of both pathogens revealed the highest disease incidence, Fusarium wilt (78.00%) and collar rot (70.00%), respectively, under the soil inoculation technique.

### 3.3 Enzymatic antioxidant assay

The enzymatic antioxidant activities of phenylalanine ammonia-lyase (PAL), catalase (CAT), and peroxidase (POD) were assessed in chickpea leaves after the application of various treatments involving *Trichoderma* spp., biochar, and pathogen inoculation. *Trichoderma* and biochar had significant effects on the enzymatic antioxidants of chickpea (Table 1). All enzyme activities were expressed as μmol substrate converted per minute per mg of protein (μmol min^−1^ mg^−1^ protein), based on a standard protein concentration of 2.5 mg/mL. Catalase activity, which plays a critical role in detoxifying hydrogen peroxide produced during oxidative bursts, showed significant variation across treatments. The highest CAT activity (0.578 μmol H₂O₂ min^−1^ mg^−1^ protein) was observed in the uninoculated control (T1), while the pathogen-inoculated control (T2) showed slightly lower activity (0.570 μmol), suggesting that pathogen stress alone induced a modest antioxidant response. Treatments T3, T4, and T5, which included *Trichoderma* application, demonstrated intermediate activity ranging from 0.521 to 0.577 μmol H₂O₂ min^−1^ mg^−1^ protein. Notably, treatments T6 to T9, which involved seed bio-priming with *Trichoderma* strain, displayed CAT activity (Table 1) between 0.517 and 0.559 μmol min^−1^ mg^−1^ protein, indicating a protective enzymatic response, likely linked to *Trichoderma*-induced systemic resistance. Similarly, PAL activity is a key enzyme in the phenylpropanoid pathway associated with lignin biosynthesis and defense signaling were significantly elevated in treatments involving *Trichoderma* or biochar amendments. The highest PAL activity (0.00249 μmol trans-cinnamic acid min^−1^ mg^−1^ protein) was observed in treatment T2 (pathogen-inoculated control). Treatments T4 and T3 showed the next highest activities, with values of 0.00241 and 0.00222 μmol trans-cinnamic acid min^−1^ mg^−1^ protein, respectively (Table 1). The lowest activity was recorded in T1 at 0.00121 μmol min^−1^ mg^−1^ protein.

**Table 1:** Effect of treatments on enzymatic activities and physiological responses of chickpea.

S No.	Treatments	CAT (μmol H₂O₂ min^−1^ mg^−1^ protein)	PAL (μmol trans-cinnamic acid min^−1^ mg^−1^ protein)	POD (μmol guaiacol min^−1^ mg^−1^ protein)	Disease incidence (%)	Germination (%)	Chlorophyll (mg/g)	Phenol (mg/g)
1.	T1	0.578^a*^	0.00121^e^	0.2436^d^	65.4 ± 2.1^a^	63.2 ± 1.8^f^	1.42 ± 0.06^f^	1.62 ± 0.07^g^
2.	T2	0.570^a^	0.00249^a^	0.0880^e^	61.7 ± 2.0^a^	65.7 ± 1.5^f^	1.51 ± 0.04^ef^	1.69 ± 0.06^fg^
3.	T3	0.577^a^	0.00222^b^	0.4724^b^	3.2 ± 0.6^f^	91.2 ± 2.0^ab^	2.13 ± 0.07^bc^	2.56 ± 0.09^bc^
4.	T4	0.559^b^	0.00241^a^	0.3044^c^	36.9 ± 1.5^c^	76.3 ± 1.6^d^	1.82 ± 0.05^de^	2.12 ± 0.08^de^
5.	T5	0.546^bc^	0.00196^c^	0.4128^b^	34.1 ± 1.7^c^	78.6 ± 1.8^cd^	1.87 ± 0.06^cd^	2.20 ± 0.06^d^
6.	T6	0.543^bc^	0.00215^bc^	0.7944^a^	28.3 ± 1.4^d^	81.4 ± 1.7^bc^	1.91 ± 0.07^cd^	2.34 ± 0.07^cd^
7.	T7	0.541^bc^	0.00228^b^	1.1468^a^	25.4 ± 1.3^d^	84.6 ± 1.6^bc^	2.01 ± 0.06^bc^	2.42 ± 0.08^bc^
8.	T8	0.521^c^	0.00224^b^	0.3588^bc^	12.1 ± 0.9^e^	93.4 ± 1.9^a^	2.26 ± 0.08^ab^	2.71 ± 0.09^ab^
9.	T9	0.517^c^	0.00220^b^	0.4476^b^	9.6 ± 0.8^e^	95.1 ± 1.5^a^	2.34 ± 0.07^a^	2.88 ± 0.08^a^

CAT, catalase; PAL, phenylalanine ammonia-lyase; POD, peroxidase.

T1: *Fusarium oxysporum* f. sp. *ciceri* (FOC), T2: *Sclerotium rolfsii* (SR), T3: *Trichoderma harzianum*, T4: FOC with biochar, T5: SR with biochar, T6: FOC with *T. harzianum*, T7: SR with *T. harzianum*, T8: FOC with biochar and *T. harzianum*, T9: SR with biochar and *T. harzianum*.

^*^Different superscript letters in a column denote significant variations among treatments according to Duncan’s multiple range test (*p* ≤ 0.05).

Peroxidase (POD) activity, often correlated with cell wall strengthening and hydrogen peroxide scavenging, followed a similar trend. The highest POD activity (Table 1) was recorded in T7 (1.1468 μmol guaiacol min^−1^ mg^−1^ protein), followed by T6, T9 than in T1; 0.2436) and T2 0.088), all of which involved either Biochar or *Trichoderma* treatments. This elevated POD activity reflects the oxidative burst and associated defense responses triggered by beneficial microbes. Overall, the observed increase in CAT, PAL, and POD activities across treatments, particularly in T6, T7, and T9, underscores the synergistic effect of *Trichoderma* spp. and organic amendments like biochar enhancing the chickpea plant’s antioxidative defense system. The results suggest that these treatments not only mitigate oxidative damage but also potentially contribute to improved plant resilience against soil-borne phytopathogens such as *Sclerotium rolfsii* and *Fusarium oxysporum* f. sp. *ciceri*.

### 3.4 Non-enzymatic antioxidant assessment

*Trichoderma* and biochar also had a significant effect on non-enzymatic antioxidants (chlorophyll and total phenol) of chickpea plants (Table 1). Chlorophyll content was highest in T9 (2.34 ± 0.07 mg/g), followed closely by T8 (2.26 ± 0.08 mg/g), indicating improved photosynthetic capacity under these treatments. In contrast, the lowest chlorophyll values were observed in control plants T1 (1.42 ± 0.06 mg/g) and T2 (1.51 ± 0.04 mg/g) (Table 1), consistent with pathogen stress and tissue degradation. Phenol content, another important marker of defense response, was significantly higher in T9 (2.88 ± 0.08 mg/g) and T8 (2.71 ± 0.09 mg/g), followed by T7 and T6. These treatments correlated well with lower disease incidence and higher POD/PAL activity, suggesting their role in reinforcing cell wall strength and inhibiting pathogen proliferation. In contrast, the lowest phenol accumulation was observed in T1 (1.62 ± 0.07 mg/g) and T2 (1.69 ± 0.06 mg/g) (Table 1).

### 3.5 Responses of chickpea to different treatments

The combination of *Trichoderma* and biochar significantly influenced the defense-related enzymes in chickpea. A bidirectional, robust interactive impact was noted between the biocontrol agent and *Fusarium oxysporum* and *Sclerotium rolfsii*, which affected the synthesis of biochemicals in chickpea (Table 1).

#### 3.5.1 Germination percentage assessment

Germination percentage improved significantly with biocontrol treatments. The highest germination was observed in T9 (95.1 ± 1.5%) and T8 (93.4 ± 1.9%), followed by T3 and T7 (91.2–84.6%), whereas T1 and T2 had the lowest values (63.2–65.7%) (Table 1), correlating with higher disease pressure and lower antioxidant protection.

#### 3.5.2 Disease assessment

Disease incidence was markedly reduced in treatments involving *Trichoderma*, especially T3 (3.2 ± 0.6%) and T9 (9.6 ± 0.8%), compared to the high incidence in control treatments T1 (65.4 ± 2.1%) and T2 (61.7 ± 2.0%) (Table 1). Treatments T6–T8 also showed significant disease suppression (12.1–28.3%), suggesting an active role of biochar and *Trichoderma* in promoting plant immunity.

**Figure 2:**
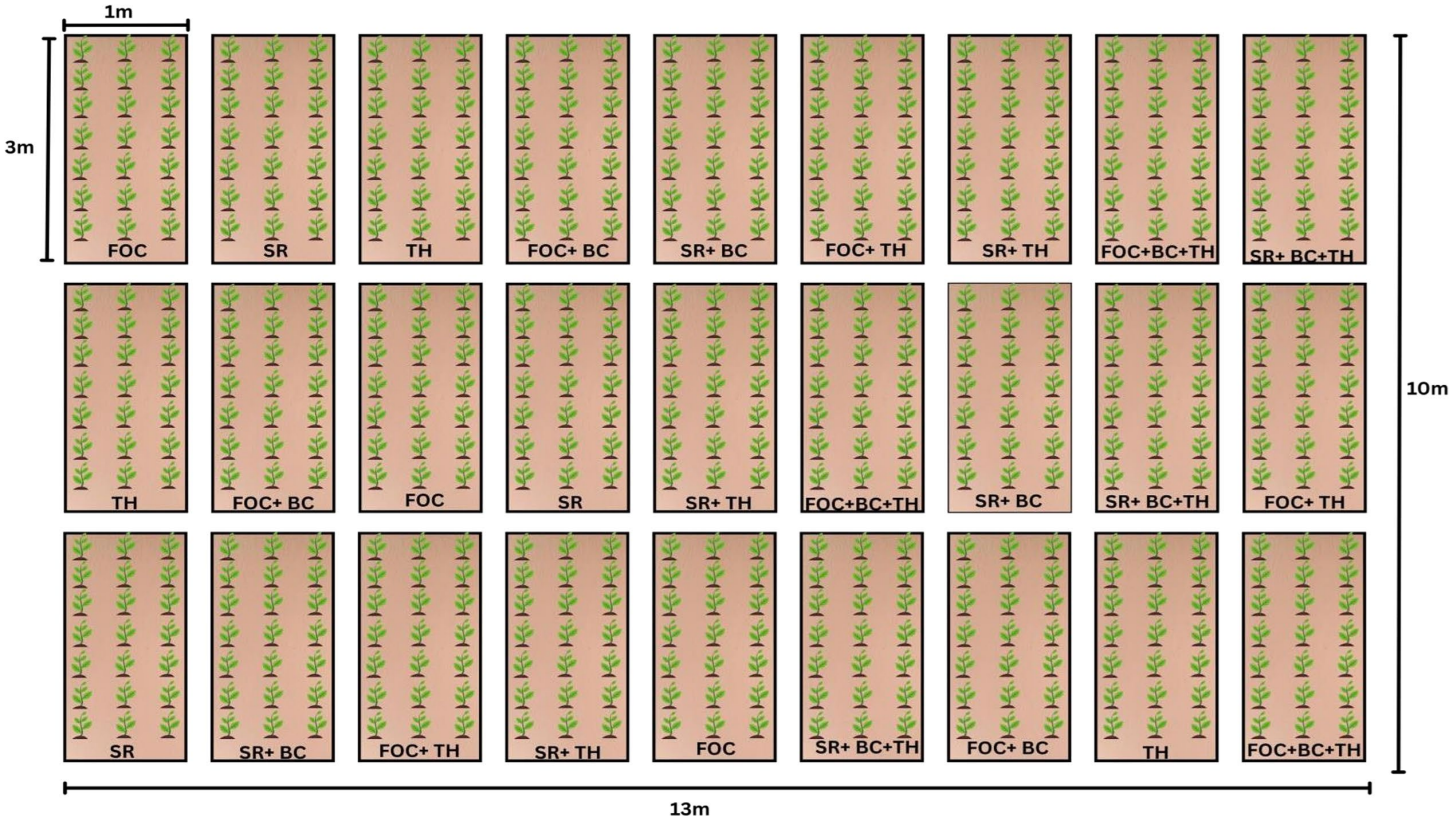
Experimental field layout for chickpea plantation during the year 2024–2025. FOC, *Fusarium oxysporum* f. sp., *ciceri*; SR, *Scelrotium rolfsii*; TH, *Trichoderma harzianum*; BC, biochar.

Together, these results demonstrate that treatments involving *Trichoderma* and biochar, particularly T9 and T8, were most effective in enhancing biochemical defense mechanisms, reducing disease severity, and improving overall plant vigor. The synergistic activation of antioxidant and phenylpropanoid pathways appear to underlie the improved stress tolerance observed in these treatments (see Figure 2).

### 3.6 Quantitative assay

#### 3.6.1 Screening of protease and chitinase activity

**Figure 3:**
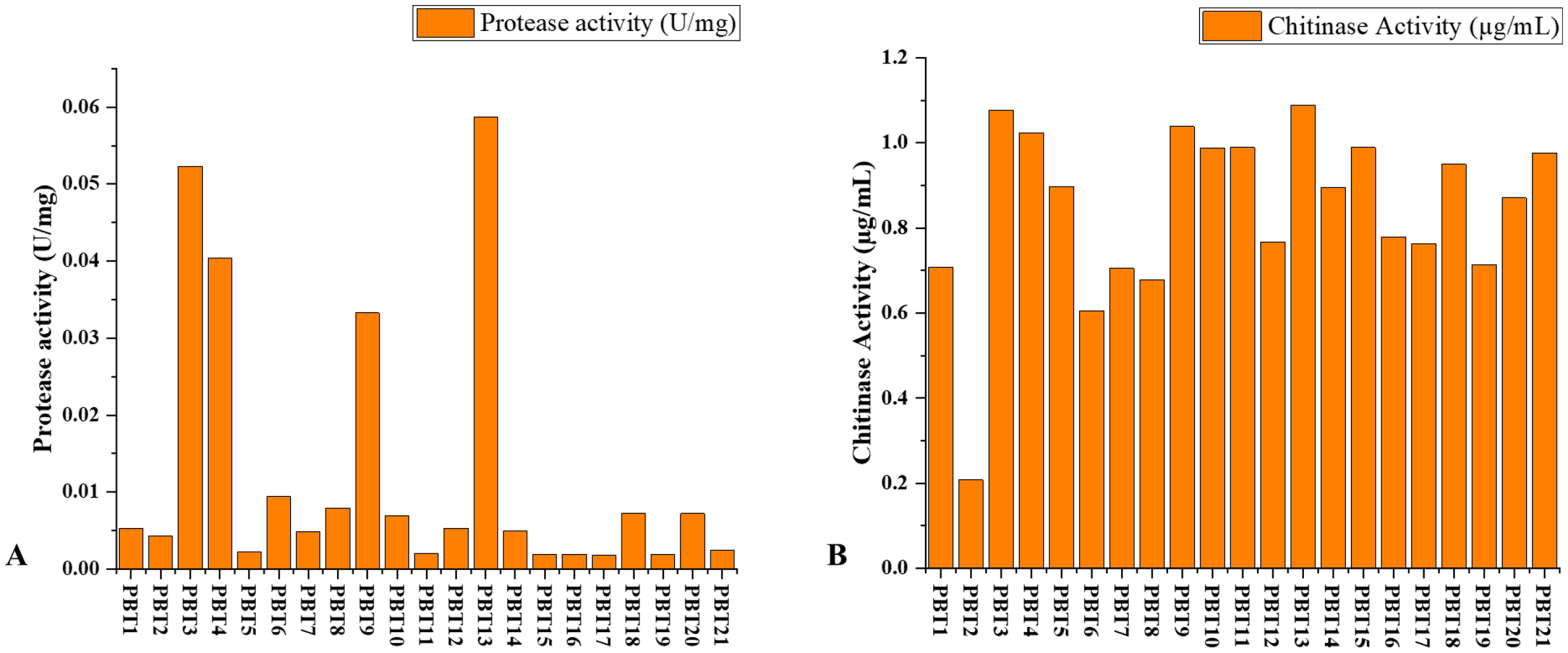
Screening of *Trichoderma* isolates for enzyme activity. **(A)** Protease activity. **(B)** Chitinase activity.

The protease and chitinase activities of *Trichoderma* isolates obtained from diverse geographical locations were assessed using both qualitative and quantitative methods. Significant variability in protease activity was observed among the isolates. The highest protease activity was recorded in PBT13 (Ludhiana) at 0.058694 U/mg, followed by PBT3 (Bathinda) at 0.052294 U/mg, and PBT4 (Amritsar) at 0.040439 U/mg. Moderate protease activity was detected in isolates PBT9 (Malerkotla) at 0.033278 U/mg, PBT6 (Fazilka) at 0.009472 U/mg, and PBT8 (Gurdaspur) at 0.00795 U/mg. In contrast, lower protease activity was observed in isolates such as PBT15 (Mansa) at 0.001906 U/mg, PBT16 (Moga) at 0.00195 U/mg, and PBT17 (Muktsar) at 0.001822 U/mg, underscoring the variability in enzyme production across different *Trichoderma* strains (Figure 3A). Similarly, chitinase activity exhibited considerable variation among the isolates. The highest chitinase activity was recorded in PBT13 (Ludhiana) at 1.089 μg/mL, followed by PBT3 (Bathinda) at 1.076 μg/mL, and PBT9 (Malerkotla) at 1.038 μg/mL. PBT4 (Amritsar) also demonstrated high chitinase activity at 1.023 μg/mL. Moderate chitinase activity was observed in PBT10 (JandK) and PBT11 (Jalandhar) at 0.988 μg/mL and 0.989 μg/mL, respectively. In contrast, lower chitinase activity was noted in isolates such as PBT2 (Barnala) at 0.208 μg/mL and PBT6 (Fazilka) at 0.605 μg/mL, indicating a reduced capacity of these strains to degrade chitin (Figure 3B). In summary, the findings reveal that specific *Trichoderma* isolates, particularly PBT13 (Ludhiana), PBT3 (Bathinda), and PBT9 (Malerkotla), exhibit robust enzymatic activity, positioning them as promising candidates for further exploration in biocontrol applications. The observed variability in enzyme production among the isolates suggests underlying differences in genetic potential and environmental adaptability. These variations warrant further investigation to evaluate their efficacy in plant disease management and their potential role in sustainable agricultural practices.

### 3.7 *In vitro* antipathogenic properties of chitinase produced by *Trichoderma harzianum*

**Figure 4:**
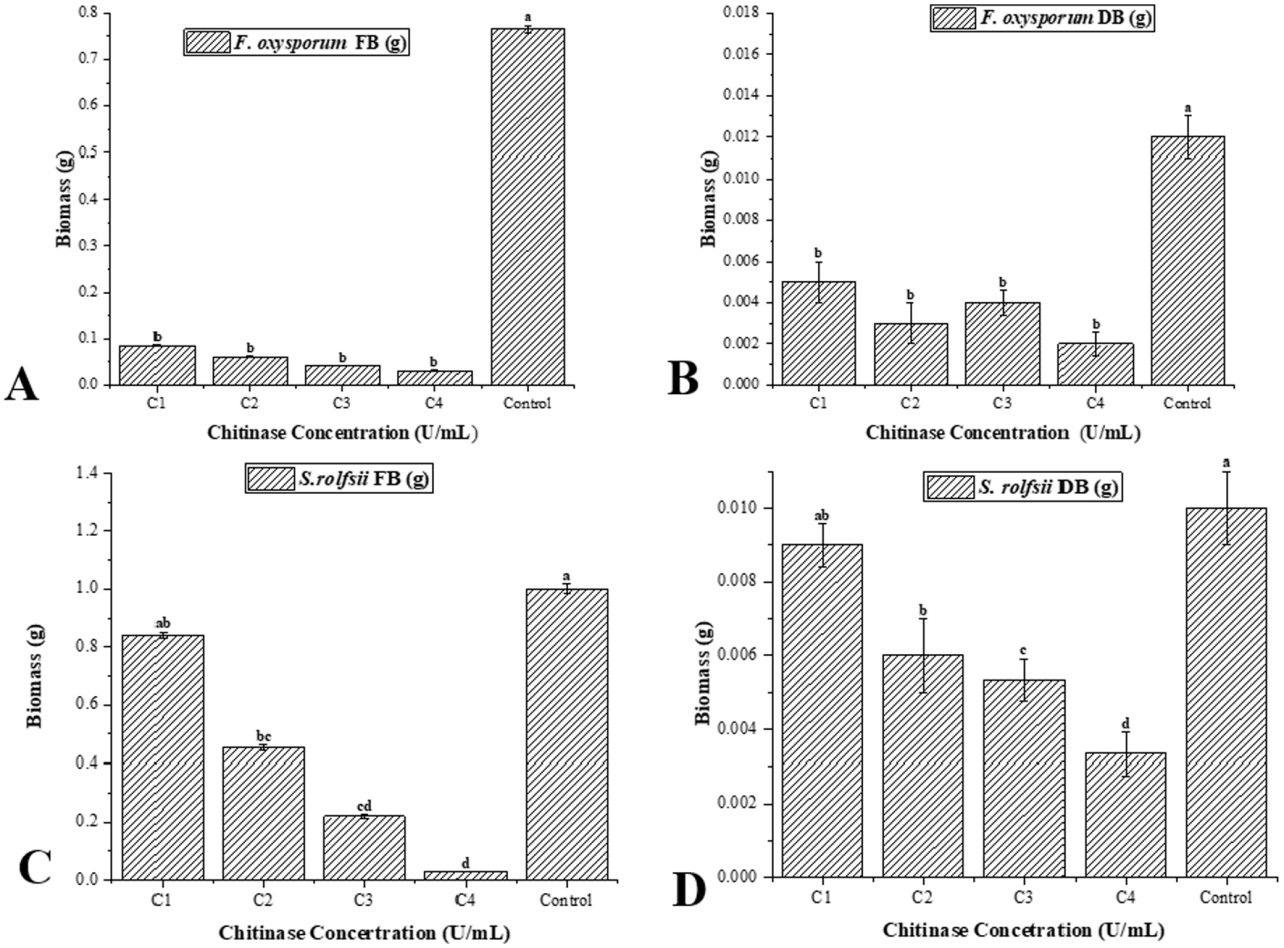
Impact of chitinase (10–60 U/mL) on biomass of pathogens **(A,B)** *F. oxysporum* and **(C,D)** *S. rolfsii*. FB, fresh iomass; DB, dry biomass.

**Figure 5:**
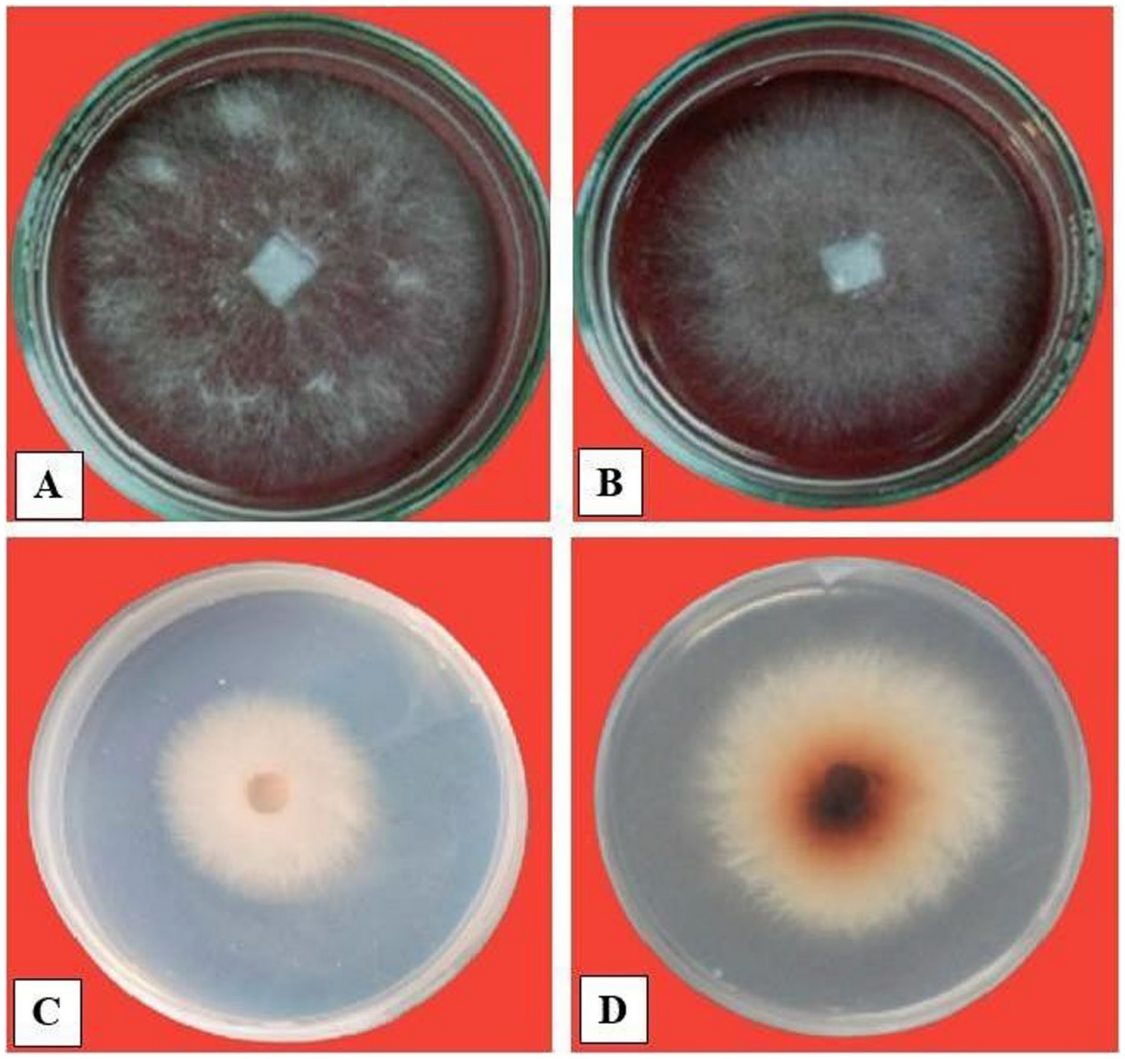
Effect of chitinase (60 U/mL) on growth of pathogens: **(A,B)** *S. rolfsii* and **(C,D)** *F. oxysporum*.

A concentrated chitinase enzyme of *Trichoderma* was used to measure its antipathogenic properties against *Sclerotium rolfsii* and *Fusarium oxysporum* f. sp. ciceri. The data and figure (Table 2 and Figures 4, 5) clearly show that chitinase efficiently suppressed the growth formation of conidia/chlamydospores and sclerotia in FOC and SR, respectively. Treatment with 10 U/mL chitinase led to a substantial reduction in fresh biomass around 88.89% for Fusarium and 16.08%% for Sclerotium. When the concentration was increased to 60 U/mL, the inhibitory effect became more pronounced, with Fusarium biomass decreasing by nearly 95.95% and S. rolfsii by approximately 97.10%. Figure 4 demonstrates a dose-dependent inhibitory effect of chitinase on both pathogens’ growth, with greater suppression observed at higher concentrations (10–60 U/mL). This observation aligns with the well-known ability of *Trichoderma* species to produce CWDEs. specifically, the chitinase produced by *Trichoderma harzianum* targets chitin, the main constituent of the fungal cell walls, leading to the degradation of hyphal tips and structural weakening. These findings support the hypothesis that *Trichoderma* disrupts pathogen integrity by enzymatically degrading their cell walls (Geraldine et al., 2013). According to Lopez-Mondejar et al. (2011), *Trichoderma* spp. secretes protease and chitinase, which contribute to the hydrolysis of host cell walls during parasitic interaction (Rukmana et al., 2020).

**Table 2:** Biomass (g) of *F. oxysporum* f. sp. *ciceri* and *S. rolfsii* after treatment with chitinase.

Chitinase (U/mL)	*F. oxysporum* FB (g)	*F. oxysporum* DB (g)	*S. rolfsii* FB (g)	*S. rolfsii* DB (g)
C1 (10)	0.085ᵇ^*^	0.008ᵇ	0.840ᵃᵇ	0.009ᵃᵇ
C2 (20)	0.061ᵇ	0.003ᵇ	0.455ᵇᶜ	0.006ᵇ
C3 (40)	0.042ᵇ	0.004ᵇ	0.220ᶜᵈ	0.005ᶜ
C4 (60)	0.031ᵇ	0.002ᵇ	0.029ᵈ	0.003ᵈ
Control	0.765ᵃ	0.012ᵃ	1.001ᵃ	0.010ᵃ

^*^Different superscript letters in a column denote significant variations among treatments according to Duncan’s multiple range test (*p* ≤ 0.05).

## 4 Discussion

*Trichoderma* is a soil-borne fungus that colonizes plant roots and directly or indirectly mediates interaction among soil, plant and environment (Tyśkiewicz et al., 2022). In recent years, the assessment of enzymatic and non-enzymatic antioxidants potential in leguminous crops has been a great concern. In the living systems, enzymatic antioxidant such as phenylalanine ammonia-lyase (PAL), peroxidase (POD) and catalase (CAT) acts as the first line of defence against oxidative stress because they have a strong and quick ability to scavenge free radicals, removing hydroxyl radicals (OH), and detoxifying hydrogen peroxide and oxygen intermediates in the cell (Kohli et al., 2019). The synthesis of the extracellular cell wall-degrading enzymes (CWDs) of *Trichoderma* has an important role in inhibition and mycoparasitism of phytopathogenic fungi (Vinale et al., 2008; Ghorbanpour et al., 2018). Moreover, the enzymes can provide several advantages to the plants by interacting with and degrading hydrocarbons and chemical pesticides used in modern agriculture (Vinale et al., 2014). It has been reported that some secreted cell wall degradation products act as elicitors, which can induce DAMP effects against phytopathogen infection (Boller and Felix, 2009). The fungal cell wall is primarily composed of *β*-glucans, mannans, chitin, and proteins (Sood et al., 2020). *Trichoderma* species are recognized as effective biocontrol agents against plant-pathogenic fungi due to their ability to produce extracellular enzymes that degrade essential cell wall components, including chitinases, −β-glucanases, and proteases (Suriani Ribeiro et al., 2019). Chitinases (lytic enzymes) secreted by *Trichoderma*, hydrolyse the glycosidic linkages between the C1 and C4 carbons in chitin, that is the principal structural component of fungal cell walls (Loc et al., 2020). Lima et al. (1999) demonstrated that the chitinase from *Trichoderma* sp. significantly impacted the cell walls of *S. rolfsii*. Similarly, El-Katatny et al. (2000) reported that the chitinase of *T. harzianum* has dramatically inhibited the growth of *S. rolfsii*. Loc et al. (2020) further confirmed the antagonistic activity of chitinase from *Trichoderma* strains against root rot pathogens. In our study, chitinase at a concentration of 60 U/mL completely repressed the *in vitro* growth of both the pathogen, *Fusarium oxysporum*, and *Sclerotium rolfsii.* In the present study, screening of *Trichoderma* isolates revealed significant variation in protease and chitinase activities, which directly correlated with their antagonistic efficacy against *S. rolfsii* and *F. oxysporum* f. sp. *ciceri*. Such enzymatic variability among isolates is echoing earlier findings (Harman et al., 2004; Loc et al., 2020), indicating that strain-specific metabolic profiles govern their biocontrol potential. These findings highlight the pivotal role of hydrolytic enzymes—particularly proteases and chitinases—in the biocontrol potential of *Trichoderma* spp. against soil-borne phytopathogens that severely affect chickpea productivity, such as *S. rolfsii* and *F. oxysporum* f. sp. *ciceri*. Among the 21 screened *Trichoderma* isolates, PBT13 (*T. harzianum*) was the most potent, exhibiting the highest enzymatic activity for both protease (0.058694 U/mg) and chitinase (1.089 μg/mL). This strong enzymatic profile directly correlated with its antagonistic activity against *S. rolfsii*, as confirmed by SEM imaging, which revealed hyphal swelling and rupture—hallmarks of mycoparasitic interaction mediated by cell wall-degrading enzymes (CWDEs) (Kumari et al., 2025). Similar morphological deformations have been recorded in *Trichoderma-*pathogen interactions (El-Katatny et al., 2000; Vinale et al., 2008), corroborating the concept that CWDE-mediated lysis serves as a principal antagonistic mechanism. Numerous studies indicate the advantageous impacts of biochar when added to soil (Patel et al., 2017; de Sousa Lima et al., 2018), and the use of *Trichoderma* promotes plant growth, boosting seed germination rate, inducing systemic defense and biological control (da Silva et al., 2017; Das, 2023). *Trichoderma* thrives in soil rich in organic compounds serving as a carbon source. Research has shown the benefits of incorporating biochar into soil (Patel et al., 2017). The use of biochar can reduce disease stress in plants (Graber et al., 2014). According to Muter et al. (2017), the treatment of biochar together with *Trichoderma* improved the germination of maize seeds and biomass production. The expression of host resistance, whether genetically determined or induced by specific treatments, is associated with various biochemical changes in plants in response to pathogen infection, particularly involving defense-related enzymes.

Peroxidase (POD) is recognized as an ISR inducer (Rahman et al., 2015; Babu et al., 2015). It is involved in defense-related responses, such as the production and removal of ROS (reactive oxygen species). Enhanced POD activity strengthens cell walls, forming a mechanical barrier agains infection (Warinowski et al., 2016; Nicholson and Hammerschmidt, 1992). Catalase acts by altering the H_2_O_2_ into water and oxygen (Murshudov et al., 1992). Additionally, it protects the plant cells against lipid peroxidation (Karanastasi et al., 2018). Our research findings indicate that CAT activity is decreased in all treatments compared to POD activity at the site of infection. A similar finding was reported by Sahni and Prasad (2020).

PAL, a key enzyme in the phenylpropanoid pathway, and a variety of secondary metabolites, such as flavonoids, lignin, and phytoalexins, are produced from l-phenylalanine and are typically linked to plant defense (Hahlbrock and Scheel, 1989; Nicholson and Hammerschmidt, 1992). The formation of phenolic compounds is linked to a high PAL level, and the early response to defense responses determines the expression of defense genes (Nicholson and Hammerschmidt, 1992). These results are consistent with earlier findings that PAL is one of the first enzymes upregulated during pathogen attack or biocontrol-mediated priming (Vidhyasekaran, 2016; Singh et al., 2022).

In our experimental findings, the combined application of *T. harzianum* with biochar substantially enhanced the physiological performance and defense-related enzymes (including CAT, POD, total phenols, and PAL), compared to individual treatments of chickpea plants under pathogen pressure. Highest concentrations of defense-related biochemicals were detected in chickpeas cultivated in soil enriched with both biochar and *Trichoderma*. The enhanced biochemical response may result from improved *Trichoderma* survival and colonization by biochar’s porous structure, which provides microhabitats and moisture retention. Similar synergistic effects have been reported by Meller Harel et al. (2012) and Graber et al. (2014), where biochar amendment improved rhizosphere conditions and stimulated systemic resistance in plants. The combined application of biochar and *Trichoderma* increased leaf CAT enzyme activity (96%) in spring corn (Amanullah and Khan, 2023). Phenolics in plants enhance the concentration of defense-related proteins, which induce structural modifications such as lignification of the cell wall, and diminishes stress caused by reactive oxygen species (Ozdal et al., 2013; Kaur et al., 2017). These findings are in consonance with earlier studies showing that *Trichoderma* spp. can enhance antioxidant enzyme activities to mitigate oxidative stress (Harman et al., 2004; Singh et al., 2020).

**Figure 6:**
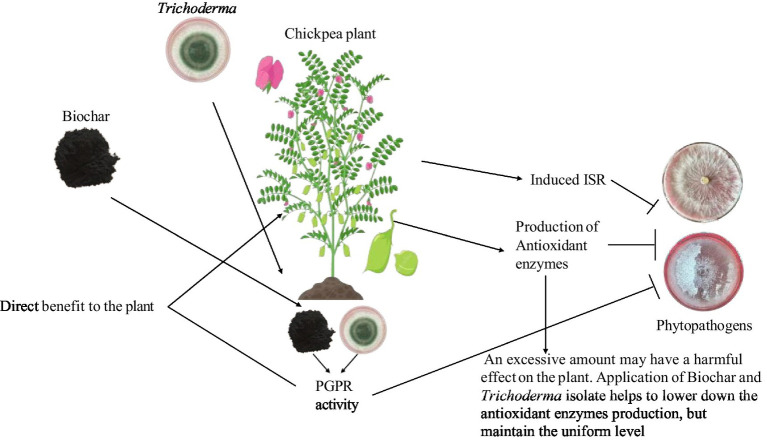
A model illustrating how soil amendment with biochar and seed biopriming with *Trichoderma* enhances antioxidant production in plants and suppresses pathogen activity.

Reports indicate that the defense of tomato plants against early blight gets enhanced by the production of phenolics and antioxidants (Awan et al., 2018). The current findings indicate that infection by *Fusarium oxysporum* and *Sclerotium rolfsii* on chickpeas in biochar-amended soil (with and without biocontrol agents) significantly affected the synthesis levels of catalase and peroxidase, thereby alleviating stress caused by pathogen infection (Attia et al., 2022). Biochar and biocontrol are recognized for their interaction with the metabolic responses of crop plants to biotic and abiotic stressors. Lalay et al. (2022) investigated the synergistic effects of biochar and biocontrol agents on the levels of chlorophyll pigments and defense-related enzymes, including catalases, in *Brassica napus* L. These findings have significantly increased our knowledge of how biochar helps develop disease resistance and convert waste materials into carbon-rich soil amendments. A strong interactive effect was found among the *Trichoderma*, biochar, and soil-borne pathogens, which influenced the biochemical production in chickpea (Table 1). *Trichoderma* regulated antioxidant production in plants and suppressed the pathogen activity (see Figure 6).

## 5 Conclusion

The synergistic treatment of *T. harzianum* and biochar significantly improved chickpea plant health, as evidenced by reduced disease incidence, increased germination rate, higher chlorophyll content, elevated antioxidant enzyme activity (CAT, POD, PAL), and increased phenol production. These results underscore the potential of integrating microbial biocontrol agents with biochar for sustainable and eco-friendly chickpea -disease management. This study demonstrates that the biocontrol efficacy of *Trichoderma* against *Fusarium oxysporum* f. sp. *ciceri* and *Sclerotium rolfsii* is strongly influenced by the release of cell wall–degrading enzymes (CWDEs), which directly damage pathogen hyphae. Among the tested isolates, *T. harzianum* (PBT13) exhibited particularly high protease and chitinase activity, key enzymes involved in suppressing soil-borne pathogens. This high enzymatic potential was closely correlated with effective pathogen inhibition, as confirmed by dual culture assays and scanning electron microscopy (SEM) analysis.

When combined with biochar—a porou organic amendment—these enzyme-active *Trichoderma* strains not only demonstrated improved survival in the soil environment but also enhanced plant defense responses. The synergistic application of *T. harzianum* (PBT13) and biochar significantly reduced disease incidence, increased germination rates, improved chlorophyll content, and elevated the activity of antioxidant enzymes (POD, PAL, CAT) and phenol production. Overall, the integration of *Trichoderma* with biochar offers a dual mode of protection through direct antagonism and induced systemic resistance, providing a sustainable and eco-sustainable method for managing soil-borne diseases in chickpea crops.

## Data Availability

The raw data supporting the conclusions of this article will be made available by the authors, without undue reservation.

## References

[ref1] Aebi H. (1984). Catalase *in vitro*. Methods Enzymol. 105, 121–126. doi: 10.1016/S0076-6879(84)05016-3 6727660

[ref2] Amanullah F. Khan W. U. D. (2023). *Trichoderma asperellum* L. coupled the effects of biochar to enhance the growth and physiology of contrasting maize cultivars under copper and nickel stresses. Plants 12:958. doi: 10.3390/plants12040958, 36840307 PMC9960312

[ref3] Ameloot N. Graber E. R. Verheijen F. G. A. De Neve S. (2014). Interactions between biochar stability and soil organisms: review and research needs. Eur. J. Soil Sci. 65, 40–52. doi: 10.1111/ejss.12064

[ref4] Attia M. S. Abdelaziz A. M. Al-Askar A. A. Arishi A. A. Abdelhakim A. M. Hashem A. H. (2022). Plant growth-promoting fungi as biocontrol tool against Fusarium wilt disease of tomato plant. J. Fungi 8:775. doi: 10.3390/jof8080775, 35893143 PMC9331501

[ref5] Awan Z. A. Shoaib A. Khan K. A. (2018). Variations in total phenolics and antioxidant enzymes cause phenotypic variability and differential resistant response in tomato genotypes against early blight disease. Sci. Hortic. 239, 216–223. doi: 10.1016/j.scienta.2018.05.044

[ref6] Babu A. N. Jogaiah S. Ito S. Nagaraj A. K. Tran L. S. P. (2015). Improvement of growth, fruit weight and early blight disease protection of tomato plants by rhizosphere bacteria is correlated with their beneficial traits and induced biosynthesis of antioxidant peroxidase and polyphenol oxidase. Plant Sci. 231, 62–73. doi: 10.1016/j.plantsci.2014.11.006 25575992

[ref7] Baek J. M. Howell C. R. Kenerley C. M. (1999). The role of an extracellular chitinase from *Trichoderma virens* Gv29-8 in the biocontrol of *Rhizoctonia solani*. Curr. Genet. 35, 41–50. doi: 10.1007/s002940050431 10022948

[ref8] Baiyee B. Pornsuriya C. Ito S. Sunpapao A. (2019). *Trichoderma spirale* T76-1 displays biocontrol activity against leaf spot on lettuce (*Lactuca sativa* L.) caused by *Corynespora cassiicola* or *Curvularia aeria*. Biol. Control 129, 195–200. doi: 10.1016/j.biocontrol.2018.10.018

[ref9] Bhuiyan K. A. (2017). Effect of *Trichoderma*-fortified compost on disease suppression, growth and yield of chickpea. Int. J. Environ. Agric. Biotechnol. 2, 831–839. doi: 10.22161/ijeab/2.2.34

[ref10] Boller T. Felix G. (2009). A renaissance of elicitors: perception of microbe-associated molecular patterns and danger signals by pattern-recognition receptors. Annu. Rev. Plant Biol. 60, 379–406. doi: 10.1146/annurev.arplant.57.032905.105346, 19400727

[ref11] Bradford M. M. (1976). A rapid and sensitive method for the quantitation of microgram quantities of protein utilizing the principle of protein-dye binding. Anal. Biochem. 72, 248–254. doi: 10.1016/0003-2697(76)90527-3, 942051

[ref12] Chance B. Maehly A. C. (1955). Assay of catalases and peroxidases. Methods Enzymol. 2, 764–775. doi: 10.1016/S0076-6879(55)02300-8 13193536

[ref13] Cherkupally R. Amballa H. Reddy B. N. (2017). *In vitro* screening for enzymatic activity of *Trichoderma* species for biocontrol potential. Ann. Plant Sci. 6, 1784–1789. doi: 10.21746/aps.2017.6.11.11

[ref14] Chowdary P. G. B. S. M. Jameema G. Charishma K. V. (2024). Collar and stem rot pathogen—*Sclerotium rolfsii*: a review. Plant Arch. 24, 67–72. doi: 10.51470/PLANTARCHIVES.2024.v24.no.1.010

[ref15] da Silva J. A. T. de Meiros E. V. da Silva J. M. Tenório D. D. A. Moreira K. A. da Silva Nascimento T. C. E. . (2017). Antagonistic activity of *Trichoderma* spp. against *Scytalidium lignicola* CMM 1098 and antioxidant enzymatic activity in cassava. Phytoparasitica 45, 219–225. doi: 10.1007/s12600-017-0578-x

[ref16] Das A. (2023). “Management of soil borne diseases of pulses (Greengram) using native *Trichoderma* isolates” in Doctoral dissertation (Bhubaneswar: Department of Plant Pathology, OUAT).

[ref17] de Sousa Lima J. R. de Moraes Silva W. de Meiros E. V. Duda G. P. Corrêa M. M. Martins Filho A. P. . (2018). Effect of biochar on physicochemical properties of a sandy soil a n d maize growth in a greenhouse experiment. Geoderma 319, 14–23. doi: 10.1016/j.geoderma.2017.12.033

[ref18] Dickerson D. P. Pascholati S. F. Hagerman A. E. Butler L. G. Nicholson R. L. (1984). Phenylalanine ammonia-lyase and hydroxycinnamate: CoA ligase in maize mesocotyls inoculated with *Helminthosporium maydis* or *Helminthosporium carbonum*. Physiol. Plant Pathol. 25, 111–123. doi: 10.1016/0048-4059(84)90050-X

[ref19] Dutta P. Mahanta M. Singh S. B. Thakuria D. Deb L. Kumari A. . (2023). Molecular interaction between plants and *Trichoderma* species against soil-borne plant pathogens. Front. Plant Sci. 14:1145715. doi: 10.3389/fpls.2023.1145715, 37255560 PMC10225716

[ref20] Elad Y. Chet I. (1995). “Practical approaches for biocontrol implementation” in Novel approaches to integrated pest management (Boca Raton, FL: Lewis Publishers), 323–338.

[ref21] El-Katatny M. H. Gudelj M. Robra K. H. Elnaghy M. A. Gübitz G. M. (2001). Characterization of a chitinase and an endo-β-1,3-glucanase from *Trichoderma harzianum* Rifai T24 involved in control of the phytopathogen *Sclerotium rolfsii*. Appl. Microbiol. Biotechnol. 56, 137–143. doi: 10.1007/s002530100646 11499921

[ref22] El-Katatny M. H. Somitsch W. Robra K. H. El-Katatny M. S. Gübitz G. M. (2000). Production of chitinase and β-1,3-glucanase by *Trichoderma harzianum* for control of the phytopathogenic fungus *Sclerotium rolfsii*. Food Technol. Biotechnol. 38, 173–180.

[ref23] Farhangi-Abriz S. Torabian S. (2017). Antioxidant enzyme and osmotic adjustment changes in bean seedlings as affected by biochar under salt stress. Ecotoxicol. Environ. Saf. 137, 64–70. doi: 10.1016/j.ecoenv.2016.11.029 27915144

[ref24] Geraldine A. M. Lopes F. A. C. Carvalho D. D. C. Barbosa E. T. Rodrigues A. R. Brandão R. S. . (2013). Cell wall-degrading enzymes and parasitism of sclerotia are key factors on field biocontrol of white mold by *Trichoderma* spp. Biol. Control 67, 308–316. doi: 10.1016/j.biocontrol.2013.09.013

[ref25] Ghorbanpour M. Omidvari M. Abbaszadeh-Dahaji P. Omidvar R. Kariman K. (2018). Mechanisms underlying the protective effects of beneficial fungi against plant diseases. Biol. Control 117, 147–157. doi: 10.1016/j.biocontrol.2017.11.006

[ref26] Gimenez E. Salinas M. Manzano-Agugliaro F. (2018). Worldwide research on plant defense against biotic stresses as improvement for sustainable agriculture. Sustainability 10:391. doi: 10.3390/su10020391

[ref27] Graber E. R. Frenkel O. Jaiswal A. K. Elad Y. (2014). How may biochar influence severity of diseases caused by soilborne pathogens? Carbon Manag. 5, 169–183. doi: 10.1080/17583004.2014.913360, 41180919

[ref28] Hahlbrock K. Scheel D. (1989). Physiology and molecular biology of phenylpropanoid metabolism. Annu. Rev. Plant Physiol. Plant Mol. Biol. 40, 347–369. doi: 10.1146/annurev.pp.40.060189.002023

[ref29] Hale L. Luth M. Crowley D. (2015). Biochar characteristics relate to its utility as an alternative soil inoculum carrier to peat and vermiculite. Soil Biol. Biochem. 81, 228–235. doi: 10.1016/j.soilbio.2014.11.023

[ref30] Hamid R. Khan M. A. Ahmad M. Ahmad M. M. Abdin M. Z. Musarrat J. . (2013). Chitinases: an update. J. Pharm. Bioallied Sci. 5, 21–29. doi: 10.4103/0975-7406.106559, 23559820 PMC3612335

[ref31] Harman G. E. Howell C. R. Viterbo A. Chet I. Lorito M. (2004). *Trichoderma* species—opportunistic, avirulent plant symbionts. Nat. Rev. Microbiol. 2, 43–56. doi: 10.1038/nrmicro797, 15035008

[ref32] Hartl L. Zach S. Seidl-Seiboth V. (2012). Fungal chitinases: diversity, mechanistic properties and biotechnological potential. Appl. Microbiol. Biotechnol. 93, 533–543. doi: 10.1007/s00253-011-3723-3 22134638 PMC3257436

[ref33] Haware M. P. Nene Y. L. Natarajan M. (1996). Survival of *Sclerotium rolfsii* and its role in collar rot of chickpea. Plant Dis. Res. 11, 46–50.

[ref34] Jaba J. Bhandi S. Deshmukh S. Pallipparambil G. R. Mishra S. P. Arora N. (2021). “Identification, evaluation and utilization of resistance to insect pests in grain legumes: advancement and restrictions” in Genetic enhancement in major food legumes: advances in major food legumes (Cham: Springer), 197–230.

[ref35] Jameel S. Hameed A. Shah T. M. (2021). Biochemical profiling for antioxidant and therapeutic potential of Pakistani chickpea (*Cicer arietinum* L.) genetic resource. Front. Plant Sci. 12:663623. doi: 10.3389/fpls.2021.663623, 33927742 PMC8076736

[ref36] Jukanti A. K. Gaur P. M. Gowda C. L. Chibbar R. N. (2012). Nutritional quality and health benefits of chickpea (*Cicer arietinum* L.): a review. Br. J. Nutr. 108, S11–S26. doi: 10.1017/S0007114512000797, 22916806

[ref37] Karanastasi E. Kostara T. Malamos N. Zervoudakis G. (2018). Catalase activity, lipid peroxidation, and protein concentration in leaves of tomato infected with *Meloidogyne javanica*. Nematropica 48, 15–20.

[ref38] Kaur H. Salh P. K. Singh B. (2017). Role of defense enzymes and phenolics in resistance of wheat crop (*Triticum aestivum* L.) towards aphid complex. J. Plant Interact. 12, 304–311. doi: 10.1080/17429145.2017.1373873

[ref39] Kohli S. K. Khanna K. Bhardwaj R. Abd_Allah E. F. Ahmad P. Corpas F. J. (2019). Assessment of subcellular ROS and NO metabolism in higher plants: multifunctional signaling molecules. Antioxidants 8:641. doi: 10.3390/antiox8120641, 31842380 PMC6943533

[ref40] Kokalis-Burelle N. Porter D. M. Rodriquez-Kabana R. Smith D. H. Subrahmanyam P. (1997). Compendium of peanut diseases. 2nd Edn. St. Paul, MN: APS Press.

[ref41] Koma B. (2023). “Predominant soil borne pathogens and integrated approach for the management of soil borne diseases” in Recent advances in agricultural sciences and technology (New Delhi: Ariana Publishers & Distributors).

[ref42] Kubicek C. P. Starr T. L. Glass N. L. (2014). Plant cell wall-degrading enzymes and their secretion in plant-pathogenic fungi. Annu. Rev. Phytopathol. 52, 427–451. doi: 10.1146/annurev-phyto-102313-045831, 25001456

[ref43] Kumar S. Gupta R. N. (2019). Effect of chickpea stunt disease on yield attributing traits and yield of chickpea. Biol. Forum Int. J. 11, 192–195.

[ref44] Kumar N. Hong S. Zhu Y. Garay A. Yang J. Henderson D. . (2025). Comprehensive review of chickpea (*Cicer arietinum*): nutritional significance, health benefits, techno-functionalities, and food applications. Compr. Rev. Food Sci. Food Saf. 24:e70152. doi: 10.1111/1541-4337.70152, 40047318

[ref45] Kumari R. Kumar V. Arukha A. P. Rabbee M. F. Ameen F. Koul B. (2024). Screening of the biocontrol efficacy of potent *Trichoderma* strains against *Fusarium oxysporum* f. sp. *ciceri* and *Sclerotium rolfsii* causing wilt and collar rot in chickpea. Microorganisms 12:1280. doi: 10.3390/microorganisms12071280 39065049 PMC11278996

[ref46] Kumari R. Kumar V. Koul R. Abul Farah M. Mishra H. (2025). Synergistic effects of *Trichoderma* and biochar on the biocontrol of two soil borne phytopathogens in chickpeas. Front. Microbiol. 16:1583114. doi: 10.3389/fmicb.2025.1583114 40376459 PMC12078217

[ref47] Lalay G. Ullah S. Ahmed I. (2022). Physiological and biochemical responses of *Brassica napus* L. to drought-induced stress by the application of biochar and plant growth-promoting rhizobacteria. Microsc. Res. Tech. 85, 1267–1281. doi: 10.1002/jemt.23993 34813127

[ref48] Landi M. Lo Piccolo E. Pellegrini E. Agati G. Giordano C. Giordani T. . (2019). The use of red species for urban “greening” in the age of climate change. Agrochimica, 157–161.

[ref49] Lehmann J. Rillig M. C. Thies J. Masiello C. A. Hockaday W. C. Crowley D. (2011). Biochar effects on soil biota—a review. Soil Biol. Biochem. 43, 1812–1836. doi: 10.1016/j.soilbio.2011.04.022

[ref50] Lima L. H. C. De Marco J. L. Ulhoa C. J. Felix C. R. (1999). Synthesis of a *Trichoderma* chitinase which affects the *Sclerotium rolfsii* and *Rhizoctonia solani* cell walls. Folia Microbiol. 44, 45–49. doi: 10.1007/BF02816220 10489693

[ref51] Liu H. Brettell L. E. Qiu Z. Singh B. K. (2020). Microbiome-mediated stress resistance in plants. Trends Plant Sci. 25, 733–743. doi: 10.1016/j.tplants.2020.03.014, 32345569

[ref52] Loc N. H. Huy N. D. Quang H. T. Lan T. T. Ha T. T. T. (2020). Characterisation and antifungal activity of extracellular chitinase from a biocontrol fungus, *Trichoderma asperellum* PQ34. Mycology 11, 38–48. doi: 10.1080/21501203.2019.1703839 32128280 PMC7033689

[ref53] Lopez-Mondejar R. Ros M. Pascual J. A. (2011). Mycoparasitism-related gene expression of *Trichoderma harzianum* isolates to evaluate their efficacy as biological control agents. Biol. Control 56, 59–66. doi: 10.1016/j.biocontrol.2010.10.003

[ref54] Lorito M. Peterbauer C. K. Hayes C. K. Harman G. E. (1994). Synergistic interaction between fungal cell wall degrading enzymes and different antifungal compounds on spore germination. Microbiology 140, 623–629. doi: 10.1099/00221287-140-3-623 8012584

[ref55] Mandal S. Mallick N. Mitra A. (2009). Salicylic acid-induced resistance to *Fusarium oxysporum* f. sp. *lycopersici* in tomato. Plant Physiol. Biochem. 47, 642–649. doi: 10.1016/j.plaphy.2009.03.001, 19329332

[ref56] Manhas R. K. Kaur T. (2016). Biocontrol potential of *Streptomyces hydrogenans* strain DH16 toward *Alternaria brassicicola* to control damping off and black leaf spot of *Raphanus sativus*. Front. Plant Sci. 7:1869. doi: 10.3389/fpls.2016.01869, 28018402 PMC5159428

[ref57] Marinari S. Masciandaro G. Ceccanti B. Grego S. (2000). Influence of organic and mineral fertilisers on soil biological and physical properties. Bioresour. Technol. 72, 9–17. doi: 10.1016/S0960-8524(99)00094-2

[ref58] Mejía C. Ardila H. D. Espinel C. Brandão P. F. Villamizar L. (2021). Use of *Trichoderma koningiopsis* chitinase to enhance the insecticidal activity of *Beauveria bassiana* against *Diatraea saccharalis*. J. Basic Microbiol. 61, 814–824. doi: 10.1002/jobm.202100161, 34312885

[ref59] Meller Harel Y. Elad Y. Rav-David D. Borenstein M. Shulchani R. Lew B. . (2012). Biochar mediates systemic response of strawberry to foliar fungal pathogens. Plant Soil 357, 245–257. doi: 10.1007/s11104-012-1129-3

[ref60] Minhas P. S. Rane J. Pasala R. K. (2017). “Abiotic stresses in agriculture: an overview” in Abiotic stress management for resilient agriculture (Singapore: Springer), 3–8.

[ref61] Mitter B. Pfaffenbichler N. Sessitsch A. (2019). Plant–microbe partnerships in 2020: microbiome diversity, functions and applications. Curr. Opin. Microbiol. 49, 63–72. doi: 10.1016/j.mib.2019.10.006 31731227

[ref62] Mohammad-Razdari A. Rousseau D. Bakhshipour A. Taylor S. Poveda J. Kiani H. (2022). Recent advances in e-monitoring of plant diseases. Biosens. Bioelectron. 201:113953. doi: 10.1016/j.bios.2021.113953 34998118

[ref63] Murshudov G. N. Melik-Adamyan W. R. Grebenko A. I. Barynin V. V. Vagin V. V. Vainshtein B. K. . (1992). Three dimensional structure of catalase from *Micrococcus lysodeikticus* at 1.5 Å resolutions. FEBS Lett. 312, 127–131. doi: 10.1016/0014-5793(92)80919-8 1426241

[ref64] Muter O. Grantina-Ievina L. Makarenkova G. Vecstaudza D. Strikauska S. Selga T. . (2017). Effect of biochar and *Trichoderma* application on fungal diversity and growth of *Zea mays* in a sandy loam soil. Environ. Exp. Biol. 15, 179–187. doi: 10.22364/eeb.15.30

[ref65] Nicholson R. L. Hammerschmidt R. (1992). Phenolic compounds and their role in disease resistance. Annu. Rev. Phytopathol. 30, 369–389. doi: 10.1146/annurev.py.30.090192.002101

[ref66] O’Gorman C. M. Fuller H. T. Dyer P. S. (2009). Discovery of a sexual cycle in the opportunistic fungal pathogen *Aspergillus fumigatus*. Nature 457, 471–474. doi: 10.1038/nature07528, 19043401

[ref67] Ozdal T. Capanoglu E. Altay F. (2013). A review on protein–phenolic interactions and associated changes. Food Res. Int. 51, 954–970. doi: 10.1016/j.foodres.2013.02.009

[ref68] Pandao M. R. Lalitha G. R. Kishore A. J. Padhan S. R. Panotra N. Gulaiya S. . (2023). A comprehensive review on biochar. Int. J. Environ. Clim. Change 13, 3453–3461. doi: 10.9734/ijecc/2023/v13i113520

[ref69] Pande S. Sharma M. Gaur P. M. Gowda C. L. Reddy M. V. (2023). Emerging fungal diseases of chickpea: distribution, yield losses and management strategies. Crop Prot. 165:106223. doi: 10.1016/j.cropro.2023.106223

[ref70] Patel A. Khare P. Patra D. D. (2017). “Biochar mitigates salinity stress in plants” in Plant adaptation strategies in changing environment (Singapore: Springer), 153–182.

[ref71] Poveda J. (2021). *Trichoderma* as biocontrol agent against pests: new uses for a mycoparasite. Biol. Control 159:104634. doi: 10.1016/j.biocontrol.2021.104634

[ref72] Punja Z. K. Jenkins S. F. (1984). Influence of temperature, moisture, modified gaseous atmosphere, and depth in soil on eruptive sclerotial germination of *Sclerotium rolfsii*. Phytopathology 74, 749–754. doi: 10.1094/Phyto-74-749

[ref73] Rahman A. Uddin W. Wenner N. G. (2015). Induced systemic resistance responses in perennial ryegrass against *Magnaporthe oryzae* elicited by semi-purified surfactin lipopeptides and live cells of *Bacillus amyloliquefaciens*. Mol. Plant Pathol. 16, 546–558. doi: 10.1111/mpp.12209 25285593 PMC6638512

[ref74] Rukmana S. Ansori A. N. Kusala M. K. Utami U. Wahyudi D. Mandasari A. A. (2020). Molecular identification of *Trichoderma* isolates from sugarcane bagasse based on internal transcribed spacer (ITS) rDNA. Res. J. Pharm. Technol. 13, 3300–3304. doi: 10.5958/0974-360X.2020.00585.5

[ref75] Sahni S. Prasad B. D. (2020). Management of collar rot disease using vermicompost and a PGPR strain *Pseudomonas* sp. and their effect on defense-related enzymes in chickpea. Indian Phytopathol. 73, 301–311. doi: 10.1007/s42360-020-00203-4

[ref76] Said O. El Bouhssini M. El Khatib A. Bouaichi A. Moumni M. Kabbaj H. . (2022). The chickpea pod borer, *Helicoverpa armigera* (Hübner): yield loss estimation and biorational insecticide assessment in Morocco. Agronomy 12:3017. doi: 10.3390/agronomy12123017

[ref77] Sandhya C. Adapa L. K. Nampoothiri K. M. Binod P. Szakacs G. Pandey A. (2004). Extracellular chitinase production by *Trichoderma harzianum* in submerged fermentation. J. Basic Microbiol. 44, 49–58. doi: 10.1002/jobm.200310284 14768028

[ref78] Sennoi R. Jogloy S. Saksirirat W. Kesmala T. Singkham N. Patanothai A. (2012). Levels of *Sclerotium rolfsii* inoculum influence identification of resistant genotypes in Jerusalem artichoke. Afr. J. Microbiol. Res. 6, 6755–6760. doi: 10.5897/AJMR12.1449

[ref79] Sharma V. Salwan R. Sharma P. N. Gulati A. (2017). Integrated translatome and proteome: approach for accurate portraying of widespread multifunctional aspects of *Trichoderma*. Front. Microbiol. 8:1602. doi: 10.3389/fmicb.2017.01602, 28900417 PMC5581810

[ref80] Sharma V. Sharma A. Salwan R. (2020). “Overview and challenges in the implementation of plant beneficial microbes” in Molecular aspects of plant beneficial microbes in agriculture. eds. Salwan R. Sharma V. (London: Academic Press), 1–18.

[ref81] Sharma B. K. Singh H. B. Singh S. P. (2002). Induction of systemic resistance in chickpea against *Fusarium oxysporum* f. sp. *ciceri* by plant growth-promoting rhizobacteria. Mycorrhiza 12, 313–319.12466919

[ref82] Shoaib A. Aslam N. Aslam N. (2013). *Trichoderma harzianum*: adsorption, desorption, isotherm, and FTIR studies. J. Anim. Plant Sci. 23, 1460–1465.

[ref83] Singh A. Singh H. B. Maheshwari D. K. (2003). Biocontrol of *Fusarium oxysporum* f. sp. *ciceri* by plant growth promoting rhizobacteria (PGPR): effect on chickpea growth and disease development. Indian J. Microbiol. 43, 81–86.

[ref84] Singh N. K. Singh M. S. Pandey R. (2022). Antagonistic activity and defense response of plant growth-promoting rhizobacteria against *Fusarium* spp. in tomato. J. Plant Interact. 17, 129–139.

[ref85] Singh A. Singh D. Singh H. B. Sarma B. K. (2019). Biopriming of chickpea seeds with *Trichoderma* for protection against Fusarium wilt. Biol. Control 130, 155–164.

[ref86] Singh V. Singh H. B. Upadhyay R. S. (2020). “Role of *Trichoderma* spp. in biotic stress management” in *Trichoderma*: agricultural applications and beyond. ed. Singh V. (Singapore: Springer), 107–123.

[ref87] Sood M. Kapoor D. Kumar V. Sheteiwy M. S. Ramakrishnan M. Landi M. . (2020). *Trichoderma*: the “secrets” of a multitalented biocontrol agent. Plants 9:762. doi: 10.3390/plants9060762, 32570799 PMC7355703

[ref88] Suriani Ribeiro M. Graciano de Paula R. Raquel Voltan A. de Castro R. G. Carraro C. B. José de Assis L. . (2019). Endo-β-1,3-glucanase (GH16 family) from *Trichoderma harzianum* participates in cell wall biogenesis but is not essential for antagonism against plant pathogens. Biomolecules 9:781. doi: 10.3390/biom9120781 31779176 PMC6995588

[ref89] Taylor A. G. Allen P. S. Bennett M. A. Bradford K. J. Burris J. S. Misra M. K. (1998). Seed enhancements. Seed Sci. Res. 8, 245–256. doi: 10.1017/S0960258500004141

[ref90] Tiwari S. Sharma B. Bisht S. Pant D. Kumar S. Tewari L. (2024). An eco-friendly approach harnessing *Trichoderma lixii* ORT2 for reducing chemical phosphatic fertilizer dependency and groundwater phosphorus management through integrated *in silico*, *in vitro* and omic studies. Groundw. Sustain. Dev. 26:101278. doi: 10.1016/j.gsd.2024.101278

[ref91] Trivedi P. Leach J. E. Tringe S. G. Sa T. Singh B. K. (2020). Plant–microbiome interactions: from community assembly to plant health. Nat. Rev. Microbiol. 18, 607–621. doi: 10.1038/s41579-020-0412-1, 32788714

[ref92] Tyśkiewicz R. Nowak A. Ozimek E. Jaroszuk-Ściseł J. (2022). *Trichoderma*: the current status of its application in agriculture for the biocontrol of fungal phytopathogens and stimulation of plant growth. Int. J. Mol. Sci. 23:2329. doi: 10.3390/ijms23042329, 35216444 PMC8875981

[ref93] Ulhoa C. J. Peberdy J. F. (1991). Regulation of chitinase synthesis in *Trichoderma harzianum*. J. Gen. Microbiol. 137, 2163–2169. doi: 10.1099/00221287-137-9-2163, 1748872

[ref94] Vidhyasekaran P. (2016). Fungal pathogenesis in plants and crops: molecular biology and host defense mechanisms. 2nd Edn. Boca Raton, FL: CRC Press.

[ref95] Vinale F. Sivasithamparam K. Ghisalberti E. L. Marra R. Woo S. L. Lorito M. (2008). *Trichoderma*–plant–pathogen interactions. Soil Biol. Biochem. 40, 1–10. doi: 10.1016/j.soilbio.2007.07.002

[ref96] Vinale F. Sivasithamparam K. Ghisalberti E. L. Woo S. L. Nigro M. Marra R. . (2014). *Trichoderma* secondary metabolites active on plants and fungal pathogens. Open Mycol. J. 8, 127–139. doi: 10.2174/1874437001408010127

[ref97] Warinowski T. Koutaniemi S. Kärkönen A. Sundberg I. Toikka M. Simola L. K. . (2016). Peroxidases bound to the growing lignin polymer produce natural-like extracellular lignin in a cell culture of Norway spruce. Front. Plant Sci. 7:1523. doi: 10.3389/fpls.2016.01523, 27803704 PMC5067304

[ref98] Wonglom P. Daengsuwan W. Ito S. I. Sunpapao A. (2019). Biological control of *Sclerotium* fruit rot of snake fruit and stem rot of lettuce by *Trichoderma* sp. T76-12/2 and the mechanisms involved. Physiol. Mol. Plant Pathol. 107, 1–7. doi: 10.1016/j.pmpp.2019.04.007

[ref99] Zwart R. S. Thompson J. P. Sheedy J. G. Li Y. P. Knights E. J. (2019). Resistance to plant-parasitic nematodes in chickpea: current status and future perspectives. Crop Pasture Sci. 70, 1035–1043. doi: 10.3389/fpls.2019.00966 PMC6689962 31428112

