# Supplementary material for: Protease and chitinase activity of Trichoderma isolates and their synergy with biochar in enhancing chickpea defense related enzymes
Source: Front Microbiol. 2025 Dec 10;16:1699251. doi: 10.3389/fmicb.2025.1699251 (PMC12727907; doi:10.3389/fmicb.2025.1699251)
Supplement: Supplementary file 1 [file Presentation_1.PPTX]

## Slide 1
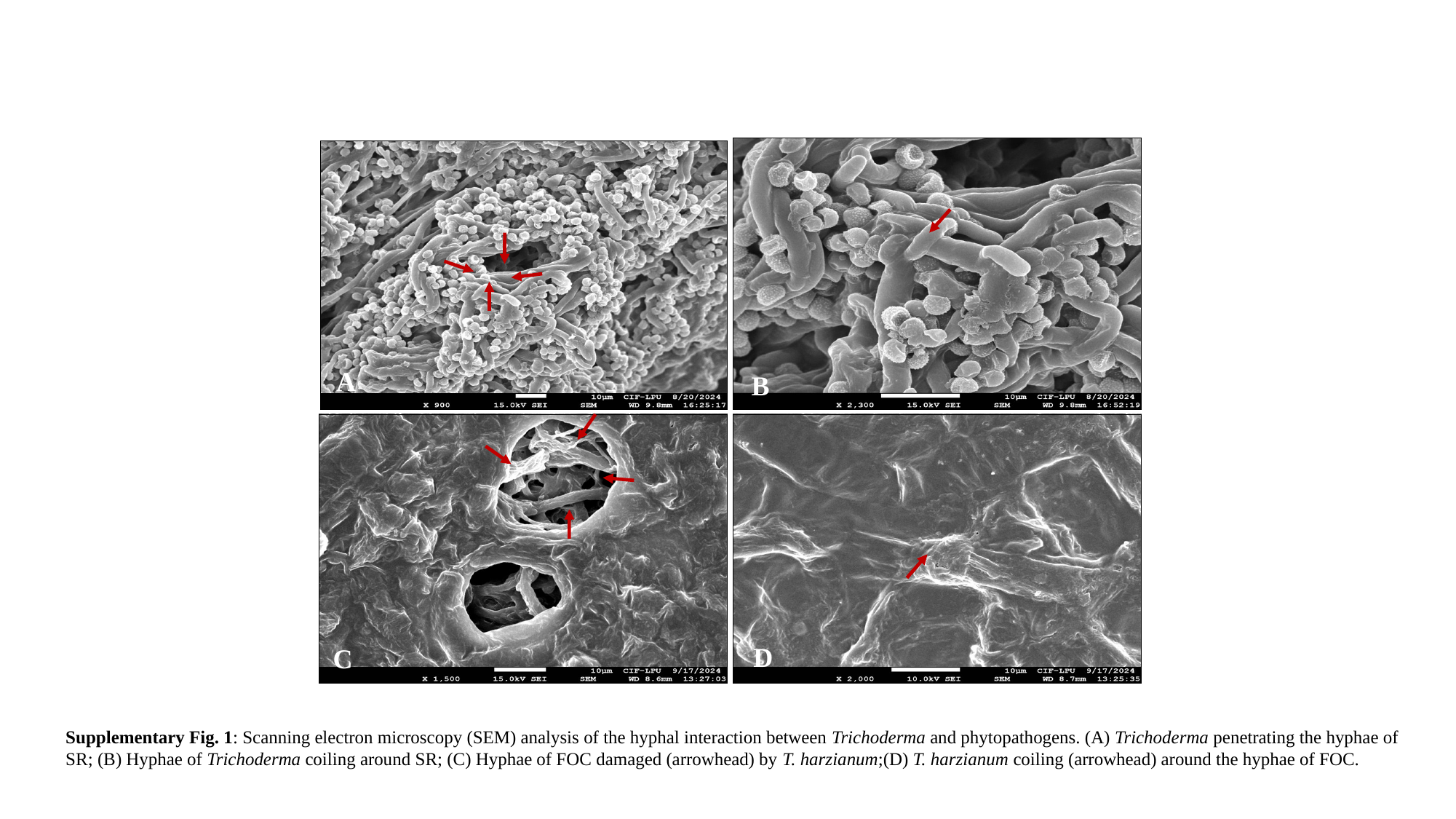

A
B
D
C
Supplementary Fig. 1: Scanning electron microscopy (SEM) analysis of the hyphal interaction between Trichoderma and phytopathogens. (A) Trichoderma penetrating the hyphae of SR; (B) Hyphae of Trichoderma coiling around SR; (C) Hyphae of FOC damaged (arrowhead) by T. harzianum;(D) T. harzianum coiling (arrowhead) around the hyphae of FOC.
